# Estimating the Age of Healthy Infants From Quantitative Myelin Water Fraction Maps

**DOI:** 10.1002/hbm.22671

**Published:** 2015-01-30

**Authors:** Douglas C Dean, Jonathan O'Muircheartaigh, Holly Dirks, Nicole Waskiewicz, Katie Lehman, Lindsay Walker, Irene Piryatinsky, Sean CL Deoni

**Affiliations:** 1 Advanced Baby Imaging Lab, School of Engineering, Brown UniversityProvidence, Rhode Island; 2 Department of Neuroimaging, King's College London, Institute of Psychiatry, Delaware Crespigny ParkLondon, United Kingdom

**Keywords:** brain development, infant imaging, myelin water fraction

## Abstract

The trajectory of the developing brain is characterized by a sequence of complex, nonlinear patterns that occur at systematic stages of maturation. Although significant prior neuroimaging research has shed light on these patterns, the challenge of accurately characterizing brain maturation, and identifying areas of accelerated or delayed development, remains. Altered brain development, particularly during the earliest stages of life, is believed to be associated with many neurological and neuropsychiatric disorders. In this work, we develop a framework to construct voxel-wise estimates of brain age based on magnetic resonance imaging measures sensitive to myelin content. 198 myelin water fraction (VF_M_) maps were acquired from healthy male and female infants and toddlers, 3 to 48 months of age, and used to train a sigmoidal-based maturational model. The validity of the approach was then established by testing the model on 129 different VF_M_ datasets. Results revealed the approach to have high accuracy, with a mean absolute percent error of 13% in males and 14% in females, and high predictive ability, with correlation coefficients between estimated and true ages of 0.945 in males and 0.94 in females. This work represents a new approach toward mapping brain maturity, and may provide a more faithful staging of brain maturation in infants beyond chronological or gestation-corrected age, allowing earlier identification of atypical regional brain development. *Hum Brain Mapp 36:1233–1244, 2015*. © **2015 The Authors Human Brain Mapping Published by Wiley Periodicals, Inc**.

## INTRODUCTION

Brain maturation encompasses a variety of structural and functional processes that respond dynamically and interdependently to learning, environment, and genetic influences. Axonal pruning, dendritic sprouting, synapse generation, and myelination begin at various stages of fetal development and continue post-natally through to adulthood [Sowell et al., 2003; Toga et al., 2006]. Cognitive and behavioral development also occurs alongside, and symbiotically with, these morphological changes [Casey et al., 2005; Johnson and Munakata, 2005]. Despite variations in the rate and extent of these processes across the population, remarkable similarities are evident within the general spatiotemporal sequence of structural and functional development [Giedd and Rapoport, 2010; Supekar et al.; 2010; Yakovlev and Lecours, 1967]. Understanding and accurately characterizing these age-related patterns is of inherent interest. Such information is critical for identifying atypical brain development believed to be associated with a growing number of neuropsychiatric disorders [Courchesne et al., 2007; Just et al., 2012], as well as providing a developmental basis for learning, language, or intellectual delay [Trauner et al., 2000].

Objective and measurable aspects of brain structure or function have been suggested as metrics of brain maturity. Recent efforts, for example, have developed frameworks for estimating “brain age” based on morphological features from inherently qualitative structural MRI scans, quantitative scans, and measures of functional connectivity. Applying a multivariate classification technique to labeled T_1_-weighted MR images of healthy adults, 56–85 years of age, Lao et al. [2004] accurately categorized 90% of subjects into 1 of 4 age-group brackets. Using a similar approach but applied to multi-contrast structural MR images of young children and adolescents, 3 to 20 years of age, Brown et al. [2012] were able to estimate individual age with a mean error of approximately 1 year by comparing 231 morphological features. Using a more quantitative approach, using measures of brain water content, Neeb et al. [2006] estimated mean brain age in healthy adults, 23–74 years of age, with a median absolute deviation of 6.3 years between real and predicted ages. More recently, measures of functional connectivity, taken as a surrogate estimate of brain network maturity, have been used to classify older children and young adults, from 7 to 30 years of age, as either children (7–11 years) or adults (24–30 years) with 91% accuracy [Dosenbach et al., 2010].

Despite the success of these approaches, significant limitations remain. These methods aggregate whole-brain age-related changes to a single representative number (i.e., the brain age), rather than estimating the age throughout the brain. This undermines both the temporal and the spatial complexity of the developmental processes taking place and makes it difficult to associate cognitive or learning delays to impaired maturation in discrete subserving brain regions. Second, none of these approaches have been implemented or tested in the earliest and most dynamic stages of brain development, that is, infancy, when impaired or altered development may have the most lasting and far reaching consequences. Early development is also believed to be when many cognitive and behavioral disorders (i.e., autism) first manifest [Courchesne et al., 2011; Wolff et al., 2012].

Prior qualitative and quantitative MRI studies of healthy infant brain development have revealed a consistent spatiotemporal pattern of evolving white and gray matter contrast that broadly mirrors the histologically established pattern of white matter myelination [Barkovich et al., 1988; Giedd et al., 1999; Paus et al., 2001]. Quantitative studies using the multicomponent relaxometry approach termed mcDESPOT [Deoni et al., 2008], which provides measures that are sensitive to the presence of myelin and reflective of the volume fraction of water trapped within the myelin sheath (the myelin water fraction [VF_M_]), also mirrors this histological sequence [Deoni et al., 2011,2012]. From mcDESPOT data acquired of male-only infants, our group has previously investigated differential growth models and has shown a Gompertz sigmoidal model faithfully characterizes the VF_M_ developmental profiles between 3 months and 5 years of age [Dean et al., 2014a]. Here, we extend this model to develop a framework for estimating the structural maturity of the brain. Using quantitative VF_M_ maps from 198 (81 female) infants, we construct a voxel-wise probabilistic model of VF_M_ maturation. This model can then be inverted to provide brain age estimates across the whole brain from any infant's VF_M_ data. This approach is tested on an additional, independent sample of 129 (57 female) VF_M_ datasets with mean absolute percent error between estimated age and actual age and reproducibility evaluated. Our results demonstrate the ability to estimate brain maturation within this early age-span, and at the voxel level, for the first time. The approach allows one to objectively examine maturation across the whole brain, potentially allowing the identification and discrimination of brain areas exhibiting accelerated or delayed growth. Such information holds intrinsic value for identifying abnormal development associated with developmental disorders, as well as in establishing structural-functional linkages that may underlie language delay or other learning disorders.

## MATERIALS AND METHODS

### Subjects

MRI data used in this study was acquired as part of a much larger, ongoing longitudinal study investigating white matter maturation [Deoni et al., 2012] and while a subset of the subject datasets have been previously used in the analysis performed in prior publications [Dean et al. 2014a,b; Deoni et al. 2012; O'Muircheartaigh et al., 2013, 2014], the analytic methods, results, and conclusions have not been previously reported in these prior publications. Parental consent was obtained in accordance with Brown University's Institutional Review Board. Enrolled children met the following inclusion/exclusion criteria. They had: (1) uncomplicated (i.e., no preeclampsia, etc., and APGAR scores > 8) singleton birth between 37 and 42 weeks gestation, (2) no familiar history or major psychiatric illness; (3) no diagnosis of major psychiatric, depressive or learning disorders; (4) no preexisting neurological conditions or major head trauma; (5) no exposure to alcohol or illicit drugs during pregnancy; and (6) no abnormalities on fetal ultrasound.

Subjects for this study consisted of 209 (86 females) healthy volunteers between 76 and 1526 days (corrected to a 40 week gestation, GC) of age at recruitment. A total of 327 successful MRI scans were acquired from these 209 subjects: 128 subjects were scanned a single time, 58 subjects were scanned twice, 21 were scanned three times, and 5 were scanned four times. Follow-up MRI scans were acquired at 6 month intervals for children under 2 years of age, and annually for children over 2 years. While males and females were found to significantly differ in birth weight, they did not significantly differ in their gestation-corrected age, gestation duration, or maternal/paternal socioeconomic status (SES; TableI).

**Table I:** Male and female group demographic information of the 209 individual subjects

Male/female subject comparison			
Characteristics	Males (123)	Females (86)	*P*-Value^a^
Gestational Corrected Age [days]	596.18 ± 415.62	549.47 ± 372.94	0.28748
Gestation Duration [weeks]	39.43 ± 1.19	39.44 ± 1.32	0.96662
Birthweight [oz]	123.34 ± 17.29	117.17 ± 14.45	**0.00085**
Maternal SES^b^	5.82 ± 1.14	5.79 ± 1.12	0.85109
Paternal SES^b^	5.65 ± 1.12	5.56 ± 1.09	0.61016

a Groups comparisons were made using a two-sample *t*-test. Correction for type 1 family-wise error was performed using the Holm–Bonferroni method.

b Maternal SES was evaluated using the Hollingshead Two Factor Index of Social Position (Miller, D.C. (1977) Handbook of Research Design and Social Measurement. New York: David McKay Company) *p* < 0.05.

### MRI Acquisition and VF_M_ Calculation

All MRI data were acquired during natural, nonsedated sleep on a Siemens Tim Trio scanner using a 12 channel radio-frequency (RF) head array [Dean et al., 2014b]. Optimized age-appropriate mcDEPSOT imaging protocols were used [Deoni et al., 2012], consisting of eight T_1_-weighted spoiled gradient echo (SPGR, spoiled FLASH) images, two inversion-prepared (IR)-SPGR images, and 16 T_1_/T_2_-weighted balanced steady state free precession (bSSFP, TrueFISP) images. SPGR and bSSFP images were acquired with incremented flip angles and the bSSFP images were acquired with two phase cycling patterns (0° and 180°).

Following acquisition and data preprocessing (including correction for possible intra-scan motion, non-brain parenchyma signal removal, and B_0_ and B_1_ magnetic field inhomogeneity correction) [Deoni 2011], voxel-wise VF_M_ maps were calculated by fitting a three-pool multicomponent relaxation model to the multiangle SPGR and SSFP data [Deoni et al., 2013]. Each participant's VF_M_ map was then nonlinearly aligned to a common, study-specific template in approximate MNI space using the Advanced Normalization Tools software package [Avants et al., 2008] and smoothed with a 3 mm Gaussian kernel. This normalization procedure has previously been shown to result in high correspondence (Pearson r correlation coefficient range of 0.90–0.99) between native and warped VF_M_ values [Dean et al., 2014a] and was, therefore, believed to reliably align individual VF_M_ maps to the study specific template.

### Calculation of Age Maps and Aggregated Brain Age

To construct the probabilistic developmental model, data from the subjects' initial MRI visit (i.e., no repeated measurements) were used. A Gompertz growth curve of the form VF_M_(age) = α*exp(−exp(*β* − *γ* × age) + *δ* × age) [Gompertz, 1825] was fit voxel-wise to the 198 (81 female) VF_M_ datasets (TableII) using the wild bootstrap [Efron, 1979] with 1000 residual resamples. Male and female data were fit independently, to avoid incorporating potential gender-based developmental differences [Lenroot et al., 2007; Lenroot and Giedd, 2010].

**Table II:** Breakdown of VF_M_ data information used for model training and testing

Training data												
	3 months	6 months	9 months	12 months	15 months	18 months	21 months	24 months	30 months	36 months	42 months	48 months
Male	21	15	11	6	5	6	6	3	15	12	7	10
Female	14	15	8	9	3	3	8	4	4	3	5	5
Age (min/max)	76/129	140/224	230/313	316/403	413/489	497/573	593/689	713/752	772/987	1020/1160	1205/1343	1366/1526
Age (mean ± std. dev.)	105.11 ± 13.54	181.47 ± 22.92	273.00 ± 24.44	357.53 ± 25.23	464.13 ± 26.83	527.78 ± 32.81	642.86 ± 31.46	729.29 ± 15.82	887.37 ± 83.46	1082.6 ± 38.78	1286.50 ± 47.37	1424.13 ± 48.37

Testing data												
	3 months	6 months	9 months	12 months	15 months	18 months	21 months	24 months	30 months	36 months	42 months	48 months
Male	4	1	13	8	11	7	9	4	5	2	5	3
Female	3	0	6	9	7	6	6	4	10	3	2	1
Age (min/max)	106/133	141/141	253/312	315/398	410/477	502/583	587/671	712/749	767/970	1079/1118	1187/1300	1351/1481
Age (mean ± std. dev.)	119.14 ± 8.83	141 ± NA	283.79 ± 16.40	364.76 ± 26.61	450.39 ± 19.37	551.00 ± 24.62	627.00 ± 28.56	728.13 ± 12.49	884.67 ± 69.22	1093.00 ± 15.86	1269.29 ± 38.82	1419.25 ± 55.09

No repeat measurements were used to train the growth model.

To invert this model and estimate brain age, a bounded golden search approach [Kiefer, 1953] was used to seek the minimum residual between the model-estimated VF_M_ value and the measured value. The algorithm was bounded by a broad age range of 30 to1800 days to ensure the optimization approach would not be biased to converge to a particular age. Calculation of age values was restricted to a central white matter mask that was created by averaging the normalized VF_M_ maps of all subjects and thresholding this average map by 0.05 to avoid regions of gray matter. Additionally, age values were only estimated for voxels that had a VF_M_ value greater than 0.015. This threshold level was specified to guarantee age values were not calculated in nonmyelinated brain regions, for example in younger participants. A global brain age was further estimated by computing the mean of the non-zero voxel-wise age estimates.

To evaluate the performance (accuracy and reproducibility) of the method, voxel-wise and global age estimates were calculated for the additional 129 (TableII) datasets not included in the model construction. Mean percent error was calculated with respect to the participants' gestation-corrected (true) age as


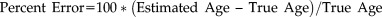
(1)

Mean global brain age estimates were compared to the true age using the mean absolute error (MAE):


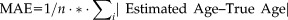
(2)

### Comparison of Global Brain Age to Developmental Age Equivalent Score

In addition to acquiring MRI data from the subjects, each child was administered the Mullen Scales of Early Learning [Mullen, 1995] at each time point. From this standardized battery age equivalent scores, defined to be the age at which the child's raw score is the median score can be computed for five separate scales (gross motor, fine motor, expressive language, receptive language, and visual reception). To examine the relationships between the global brain age estimates presented here and these similar developmental age scores, we created an overall developmental age score by averaging four of the five age equivalent scores (fine motor, expressive language, receptive language, and visual reception) and performed linear regression between this mean developmental score and estimates of global brain age. Gross motor age equivalent scores were not included in the overall developmental score as this measure is only calculated for children 0–33 months of age and, therefore, was not collected for each child. Maps and histograms of percent error, calculated between voxel estimates of age and the mean developmental age score, were also computed to examine how well age scores compare to established measures of “developmental age.”

## RESULTS

**Figure 1:**
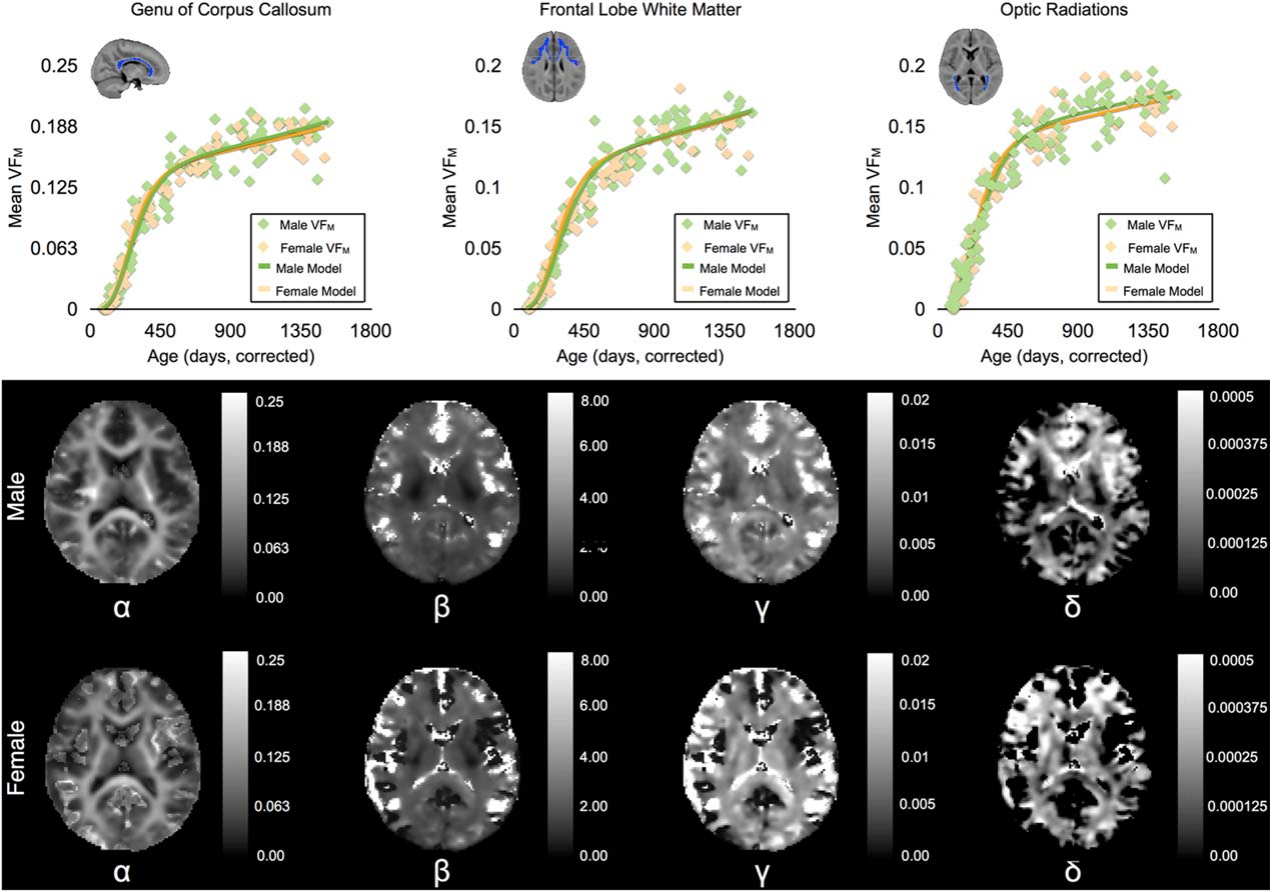
(Top Row) VF_M_ trajectories from the corpus callosum, frontal lobe white matter, and optic radiations fitted with the Gompertz growth curve for both males and females. These representative trajectories and fits illustrate how well the Gompertz model characterizes VF_M_ development. (Middle and Bottom Row) Representative male and female axial slices of Gompertz parameter volumes obtained after fitting VF_M_ values voxel-wise. Note the change in colorbar scale of these parameter maps. These parameter maps characterize the developmental growth at the voxel level and can be used to reconstruct population-averaged VF_M_ maps.

To illustrate the nonlinear development of VF_M_ during the investigated age range, representative developmental trajectories with the corresponding fitted Gompertz function for the corpus callosum, frontal white matter, and optic radiations are shown in the top row of Figure 1 (top row). These anatomical regions were defined by superimposing coregistered masks from the MNI adult template [Mazziotta et al., 2001] to the individual VF_M_ maps used in the training of the Gompertz model. Average VF_M_ values were extracted from the non-zero voxels of the representative regions and plotted with respect to the subjects' gestationally corrected age. Qualitatively, these representative trajectories highlight the observed sigmoidal pattern of development previously reported [Deoni et al., 2012; Dean et al., 2014a]. Moreover, characteristic differences in the shape of the individual regional trajectories illustrate that development, and particularly myelination, does not take place at the same time or at the same rate.

Maps corresponding to voxelwise estimates of the four free Gompertz model parameters for the male and female models are also shown in Figure 1 (middle and bottom row). Of the four parameters, *α*, controls for the overall scale and shape of the growth curve; *β* describes the lag period before the initial inflection point, and *γ* and *δ* governs the growth rate of the model.

**Figure 2:**
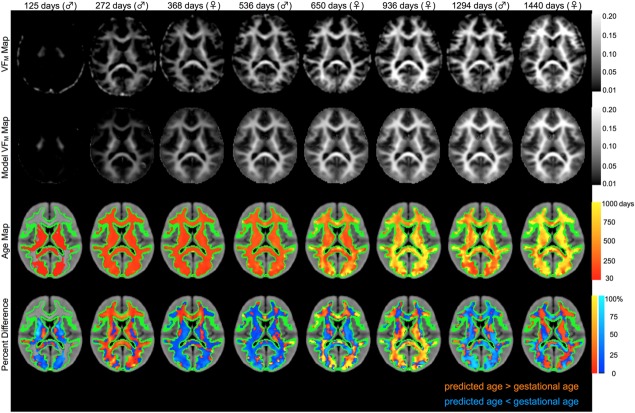
Representative axial slices of measured VF_M_, model VF_M_, age, and percent error maps for male and female subjects. Age maps and percent error maps are overlaid on a T_1_-weighted study template. Age values were calculated at voxels that had a VF_M_ value greater than 0.015 within a custom white matter mask, created by thresholding the group mean VF_M_ map at 0.02. The outline of the mask is highlighted in green. Percent error values were calculated between the predicted age values and the “true” gestationally-corrected age.

**Figure 3:**
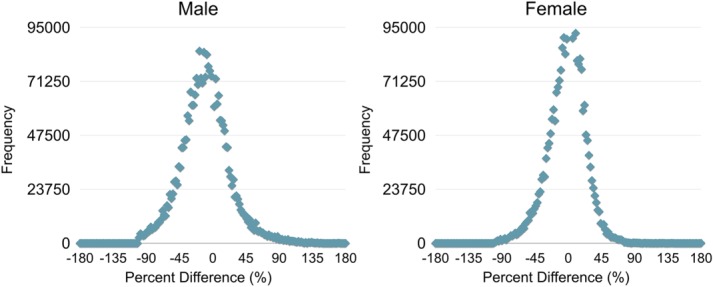
Histogram plots of the frequency of percent error values obtained from the maps of percent error between gestational corrected age and predicted age measurements. The average absolute percent error was found to be 13.36% for males and 14.63% in females.

Representative matched axial slices through acquired and model-reconstructed VF_M_ maps, estimated brain age map, and percent error maps are shown in Figure 2 for 3, 9, 18, and 42 month-old male infants, and 12, 21, 30, and 48 month-old female infants. Voxels of the age maps revealed increasing image intensity with age, directly corresponding to and in agreement with the maturation of white matter as viewed by the spatial timecourse of the acquired VF_M_ maps. While voxels of percent difference are observed to have a wide distribution across the brain, ranging from −98% to 172% and −99% to 232% for males and females, respectively, these were uniformly distributed about zero, as shown by the histograms in Figure 3. Averaged across all 72 male and 57 female test participants, the average percent error was 13.36% for males and 14.63% for females.

**Figure 4:**
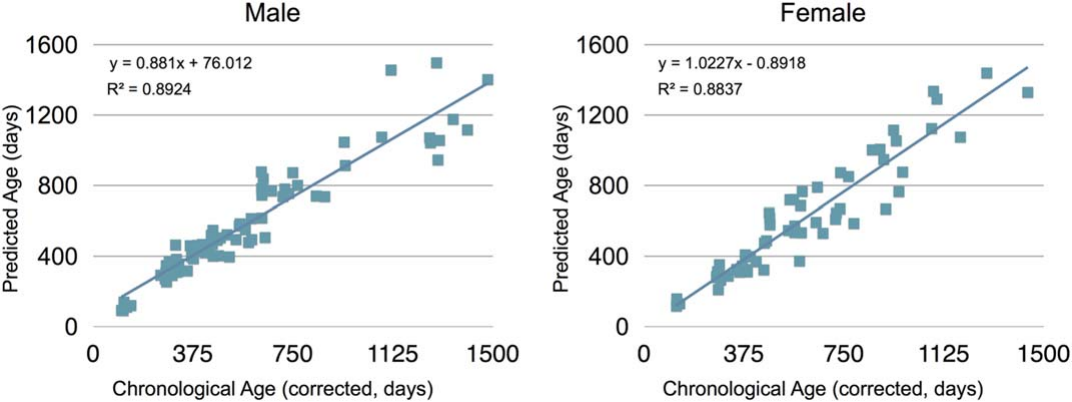
Scatter plots of the estimated brain maturation for male (left) and female (right) test datasets. Gestational-corrected age is shown on the abscissa axis and the predicted age is shown on the ordinate axis. The overall correlation between estimated age and actual age was *r* = 0.945 for males and *r* = 0.94 for females.

The mean global brain age for each subject was calculated from the voxelwise age estimates and was subsequently plotted against the subject's gestation-corrected chronological age (illustrated in Fig. 4). The correlation coefficient calculated for the males and females were 0.945 and 0.94, respectively, further demonstrating the accuracy of the method. For males, the method accounted for 89% of the variability of individual differences in age and had a MAE of 79.06 days. For females, the framework accounted for 88% of the variability of individual differences and had a MAE of 90.02 days (TableIII).

**Table III:** Performance measures of age estimation in males and females

	Best fit line	Mean absolute error	Mean percent error	Correlation coefficient
Males	1.0128^*^x − 14.613	79.06	13.36	0.9447
Females	0.8642^*^x + 71.418	90.02	14.63	0.9401

**Figure 5:**
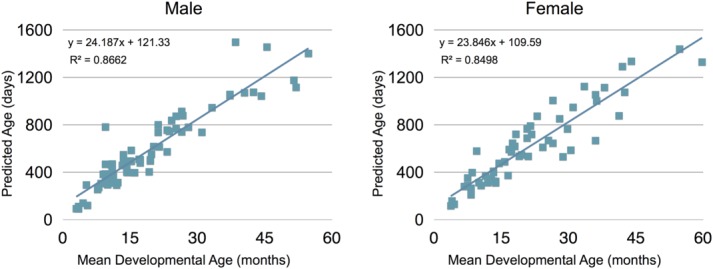
Scatter plots of the estimated brain maturation versus the mean age equivalent score (calculated from the Mullen's Scale of Early Learning measures) for male (left) and female (right) test datasets. The mean developmental age score is shown on the abscissa axis, and the predicted age is shown on the ordinate axis. The overall correlation between estimated age and actual age was *r* = 0.93 for males and *r* = 0.92 for females.

**Figure 6:**
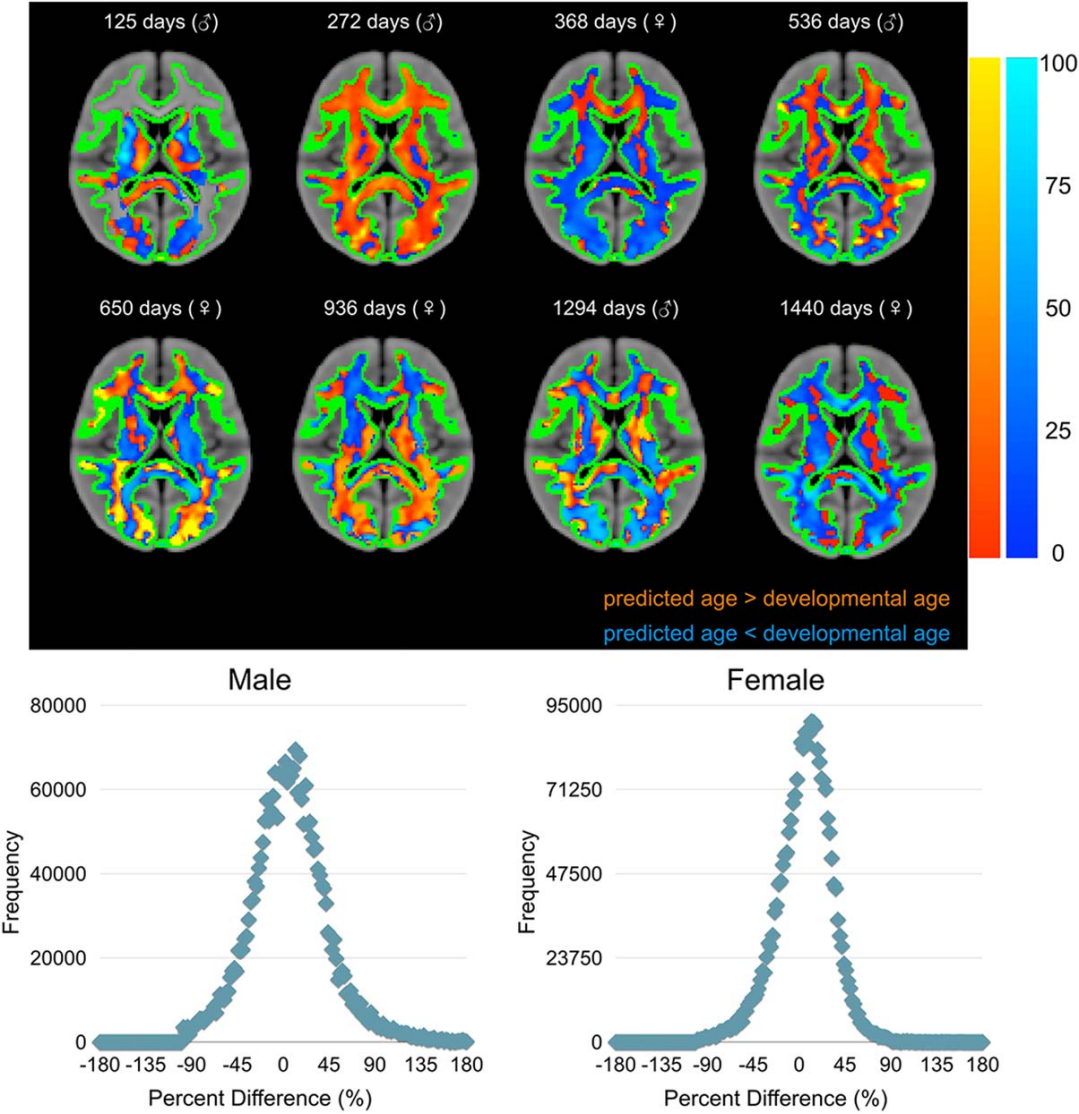
Maps of percent error calculated between the overall developmental age equivalent score and predicted age measurements. Histogram plots of the frequency of percent error values reveal the distribution of percent error values.

Average Mullen standardized (*T*-) scores of male and female subjects are given in TableIV. These measures are normalized to have a mean of 50 and standard deviation of 10. No statistical differences were found between this population mean and our sample mean in performing a *z*-test (TableIV), thus demonstrating our sample to be representative of a typical population of young children. Figure 5 shows the mean developmental age score (Mullen age equivalent scores) plotted against the global estimate of brain age for both males and females. The overall developmental age score was found to be highly correlated to the predicted global brain age with correlation coefficients of 0.93 and 0.92 for males and females, respectively. The framework accounted for 87% and 85% of the variability of individual differences in mean developmental age score, for males and females, respectively. Representative percent difference maps and histograms of percent error between the voxel age estimates and mean developmental age equivalent scores are shown in Figure 6. Similar to the comparisons with gestational age, a wide spread of percent difference values (ranging from −96% to 178% for males and −98% to 160% for females) were found between predicted and developmental age across the brain. However, these were additionally uniformly distributed about zero.

**Table IV:** Average and standard deviation of Mullen standardized (*T*-) scores for males and females

	Training data (mean ± s.d) (min – max)	*P*-value	Testing data (mean ± s.d) (min – max)	*P*-value
Males	48.03 ± 11.75 (20–80)	0.9804	48.11 ± 12.60 (20–79)	0.9321
Females	50.28 ± 11.31 (20–80)	0.4019	51.25 ± 11.75 (20–80)	0.1735

Values are computed by averaging individual *T*-scores of visual reception, expressive language, fine motor, and receptive language. No statistical differences between the population used here and a typical population, as defined by the Mullen Scales of Early Learning, were observed.

## DISCUSSION

This work sought to evaluate the utility of white matter and VF_M_ imaging in accurately characterizing the maturing brain and myelinated white matter. The method was found to perform well within the bounded age range from 3 to 48 months of age, accounting for 89% and 88% of the variance between estimated and gestation-corrected chronological age in males and females, respectively. These values compare favorably to other proposed methods in children, adolescents [Franke et al., 2012] and adults [Ashburner, 2007; Franke et al., 2010; Lao et al., 2004].

Two important distinctions exist between the VF_M_-based approach described herein and other prior techniques. First, alternative designs have typically utilized multivariate classification techniques [Brown et al., 2012; Dosenbach et al., 2010; Lao et al., 2004], where information about the age-related changes from multiple brain regions are combined into a model and used to predict a single estimate of age. Such studies are limited in the sense that they may not be able to recognize where in the brain age-related changes are occurring. Our approach, in contrast, models the developmental changes voxel-wise and thus encodes spatial information that can be used to estimate measures of maturity at a voxel-wise level. As differing brain regions grow and mature at different times throughout life [Lebel and Beaulieu, 2011], voxel-wise age estimates would allow one to observe the timing and location of these age-related changes and, therefore, may be useful at examining and monitoring neurological diseases and/or developmental disorders that are thought to alter the brain in time.

Second, we have examined a much younger cohort than prior studies. Investigations of early brain development are of increasing importance, as identification and treatment of abnormalities at the earliest stages are believed to provide the best possible outcomes. However, this development period is also one of the most dynamic, with the white matter maturation profile shown to be highly nonlinear [Dean et al., 2014a; Deoni et al., 2012; Giedd and Rapoport, 2010; Lebel and Beaulieu, 2011].

Voxel-wise estimates of age represent an unique method to examine the time course of brain maturation. As areas of the brain develop at different rates and at different times [Gao et al., 2008; Toga et al., 2006], voxel intensities that correspond to age could indicate areas that are different from what would be expected based on the child's gestation-corrected age. As shown by the histograms in Figure 3, percent error values at the voxel level ranged from −98% to 172% and −99% to 232% for males and females, respectively. While this range represents a large distribution of values, the majority of voxels were contained within ±50% of the true gestationally corrected age. This error may seem large, however, the average percent difference for males and females were 13.36% and 14.63%, respectively, corresponding to an average variation of 2.65 months for males and 2.68 months for females. The variability observed in these voxel-wise estimates is most likely due to the individual variation from the population-averaged Gompertz growth model. Improved characterization of the underlying growth model, for example, by modeling longitudinal development with nonlinear mixed models [Xu et al., 2008], may reduce the observed variability and improve age estimates at the voxel level.

While the maturation of myelin is thought to follow a similar spatial-temporal pattern of cognitive and behavioral development [Casey et al., 2005; Fields, 2008], the association of these structural and functional aspects remains poorly understood. Here, we examined the relationship between age equivalent behavioral scores and brain age estimates calculated from VF_M_ images. We found global brain age to be strongly correlated with the mean age equivalent score. These results compliment previous investigations that have shown myelination to correlate with various aspects of functional development including the development of language [Aslin and Schlaggar, 2006; Brauer et al., 2011; Pujol et al., 2006], motor skills [Shafir et al., 2008], and reading abilities [Deutsch et al., 2005; Klingberg et al., 2000]. However, although these results support the notion of concomitant structural and functional development, further investigation is needed. For example, although cross-sectional studies are informative, integration of longitudinal VF_M_ and neurobehavioral measurements may provide more meaningful inferences of the relationships between brain and cognitive development. Furthermore, the use of statistical mediation models [Baron and Kenny, 1986] may help elucidate the underlying mechanisms that relate myelination and behavioral development.

A limitation of this study, however, is the restrictive age range of the investigated sample. Although neurodevelopment during the first few years of life is indeed a period of rapid maturation, adolescence and adulthood represent a time of continuing developmental changes both in structure and function. Extension of the developed framework to incorporate older age ranges would be valuable given the potential usefulness of mapping brain age in neurodegenerative diseases such as Alzheimer's and other forms of dementia [Franke and Gaser, 2012]. To extend this approach to older populations, investigations of the Gompertz model used to characterize the underlying maturational process are needed to ensure this model remains appropriate for describing the developmental trajectory in these older populations or whether additional modifications and/or models are needed.

An additional limitation to this study is that our conclusions are limited to data acquired from a single 3.0 T MRI scanner. Although the beauty of using quantitative maps to estimate brain age over structural and functional MRI methods relies on the notion that quantitative imaging separates out scanner-dependent effects (acquisition strategy, scanner hardware), allowing for a standardized basis for comparison [Weiskopf et al., 2013], further studies investigating the reproducibility of mcDESOT across different scanners and field strengths are needed. The mcDESPOT technique has been shown to have high intrasite and intersite reproducibility [Deoni et al., 2009], however, none of the data analyzed in this work has been separately acquired at a different imaging facility, scanner, or field strength. Additional work investigating the reproducibility of the mcDESPOT imaging technique as well as the described age mapping technique on data acquired from other scanners and at different field strengths are particularly necessary to examine the generalizability of the described approach.

Although scans from the same subject were not used in the training of the developmental growth model, they were used in the testing dataset. These repeated measures may introduce a level of bias into the results presented here, and therefore, greater care would be required to model the observed developmental patterns. Longitudinal analysis techniques, including linear and nonlinear mixed models, provide the means to characterize observed patterns of change from repeated measures and have been demonstrated to be successfully implemented in studies examining brain development [Sadeghi et al. 2013]. Advanced modeling frameworks, such as these, may be advantageous in describing the nonlinear growth pattern of VF_M_ and provide a better estimation of brain age; however, examination of the histograms of percent differences reveal the values to be normally distributed about zero, giving us confidence that the inclusion of data collected at different times from the same subject is justifiable. Nonetheless, executions of longitudinal mixed modeling algorithms are highly desirable and would provide a more complete characterization of developmental patterns.

Although the presented technique offers a simplistic approach to estimating the brain age, the inclusion of additional information may provide improved accuracy. For instance, alongside VF_M_ values, mcDESPOT also provides water-pool specific T_1_ and T_2_ estimates, as well as more conventional single-component *T*_1_ and *T*_2_ values [Deoni et al., 2003]. We and others have shown *T*_1_ and *T*_2_ values to decrease exponentially throughout infancy and early childhood, and then to increase gradually throughout adulthood [Deoni et al., 2012; Hasan et al., 2010]. Incorporating these additional measures into a more complete model of brain development may improve the performance of the outlined method.

## CONCLUSION

As neurobehavioral and neuropsychiatric disorders are increasingly examined within the context of abnormal development, the ability to accurately gauge regional brain maturity becomes ever more important. Here, we have outlined a framework for age-mapping the brain using mcDESPOT-derived VF_M_ maps in healthy infants and toddlers and shown, for the first time, voxel-wise estimates of brain age that correlate strongly with gestation-corrected chronological age and average developmental age equivalent measures in healthy infants and toddlers. The method provides an objective examination of the developing brain, potentially permitting areas of accelerated or delayed maturation to be readily identified. Such information is useful within a research context toward understanding linkages between evolving neurodevelopment and malbehavior, as well as clinical-relevance in premature infants, or infants exposed to alcohol or illicit substances in utero. Although additional work is required to evaluate the utility of the method in these at-risk populations, the high accuracy and robustness of the method demonstrated in healthy infants strongly intimates its feasibility and success.
